# Supplementary material for: A single-cell time-lapse of mouse prenatal development from gastrula to birth
Source: Nature. 2024 Feb 14;626(8001):1084–93. doi: 10.1038/s41586-024-07069-w (PMC10901739; doi:10.1038/s41586-024-07069-w)
Supplement: Supplementary file 1 — This file contains Supplementary Notes 1 and 2, legends for Supplementary Tables 1–27, and Supplementary References. [file 41586_2024_7069_MOESM1_ESM.pdf]

nature portfolio

https://doi.org/10.1038/s41586-024-07069-w

Supplementary information

A single-cell time-lapse of mouse prenatal development from gastrula to birth

In the format provided by the authors and unedited

A single cell timelapse of mouse prenatal development, from gastrula to birth

Supplementary Notes

Supplementary Note 1. The Timing and Trajectories of Retinal Diversification

In mouse development, neural epithelium arises as early as E9, giving rise to ciliary and pigment epithelium as well as multipotent retinal progenitor cells (RPCs) by E10. Through the remainder of fetal development and continuing postnatally, RPCs give rise to seven major types of retinal neurons in a conserved order[1]. Here we sought to leverage the depth and temporal resolution of these data to more precisely define the developmental intervals and rates at which retinal cell types emerge, proliferate and diversify.

We re-embedded and re-annotated 160,834 cells with relevant preliminary annotations across all timepoints (**Extended Data Fig. 9a-c**). We observe that eye field is already detectable in our earliest embryo (early head fold stage; 0 somite embryo in E8.5 bin; *Pax2*+, *n* = 782 cells), diversifying towards retinal progenitors (as early as E9.75) and retinal pigment epithelium (RPE) (as early as E10), as well as a third branch that appears as early as E9.5, sharply downregulates *Rax*, and ceases proliferating by E14.5, likely corresponding to the optic stalk (**Extended Data Fig. 9d-e**). This branch is undetectable in later time points, but pathway analysis suggests this is due to terminal differentiation in the context of a rapidly growing embryo, rather than apoptosis. Among retinal neurons, differentiation towards retinal ganglion cells (RGCs) begins as early as E11.75, and towards cone photoreceptors as early as E13. As development progresses, we observe a succession of retinal neuron types appearing in the expected order (**Extended Data Fig. 9f-h**), except for Müller glia, which emerge postnatally[2]. Among RPCs, the succession of sampled timepoints fills out a continuum of transcriptional states associated with diversification towards most major retinal neuron types (**Extended Data Fig. 9a, f**)[3]. In contrast, the ciliary marginal zone (CMZ), identified as early as E11.25, remains most similar to a rapidly expanding pool of naive retinal progenitors. Strikingly, the CMZ appears to give rise to a second wave of pigment epithelium in the perinatal period, entirely separated in terms of both its transcriptional trajectory and timeframe from the branch leading to RPE, likely corresponding to the iris pigment epithelium (IPE; **Extended Data Fig. 9a, i-j**).

Reanalyzing RGCs, we identify 15 clearly distinguishable subtypes, mainly diversifying in late gestation and well-defined by specific combinations of TFs (**Extended Data Fig. 9k-l**). This extent of detected RGC diversity is on par with expectation for P0[4], suggesting that the improved performance of sci-RNA-seq3 has substantially improved its ability to discriminate neuronal subtypes.

Supplementary Note 2. The MNN approach used for graph construction is robust to subsampling and the choice of k parameter

To evaluate whether our approach to reconstructing a cell type tree is robust to technical factors or parameter choices, we took the following three approaches.

First, we examined whether the MNNs that we identified between different cell types were enriched for cells from the same embryo. Since the data from pre-gastrulation and gastrulation were generated from pooled samples, we only investigated this phenomenon for later stages, i.e. E8-P0 data generated via sci-RNA-seq3. Overall, we found that only 16.4% of MNNs from different cell types were between cells from the same embryo. However, we notably only profiled one embryo for most timepoints, which may inflate this value relative to what it might have been if we had profiled multiple embryos per timepoint. This is supported by the fact that when we look at windows with multiple embryos profiled per timepoint (E8-E10 and E13-E13.75), the proportion of MNNs from different cell types that connect cells from the same embryo was only 10.5% for E8-E10, and only 2.4% for E13-E13.75 (**Extended Data Fig.11a**). Overall, the fact that MNNs spanning cell types overwhelmingly connect cells from different embryos (and different timepoints) is reassuring.

Second, to assess the robustness of MNNs to cell sampling, we randomly subsampled 80% of cells from each developmental system during organogenesis & fetal development (except for notochord, which is a relatively rare cell type). We then repeated our MNN approach on the subsamples and compared the resulting numbers of MNNs obtained for each edge to those obtained when using the full dataset. This process was repeated 100 times for each developmental system. The resulting correlation coefficients ranged from 0.92 to 0.99, with an average of 0.98 (**Extended Data Fig.11b**). This suggests that the MNNs we identified are robust to cell sampling.

Third, we investigated the impact of our choice of k parameter in using kNNs to identify MNNs between cell types. The original k value was selected based on the log2-transformed median number of cells across cell types (k = 10 neighbors for pre-gastrulation and gastrulation subsystems, k = 15 for organogenesis & fetal development subsystems). To determine the effect of k parameter choice on the MNNs identified between cell types, we examined different k values (k = 5, 10, 20, 30, 40, 50) for kNN to identify MNNs for each developmental system during organogenesis & fetal development. We then compared the results to the original result, which was based on k = 15. The resulting Spearman correlation coefficients ranged from 0.92 to 0.99, with an average of 0.98 (**Extended Data Fig. 11c**). This suggests that the MNNs we identified are robust to the choice of k parameter.

Supplementary Tables

Supplementary Table 1. Summary of individual embryo samples.

Supplementary Table 2. Quality summary of each experiment (after filtering out low-quality cells or potential doublets).

Supplementary Table 3. Estimating the number of cells in a mouse embryo as a function of developmental stage.

Supplementary Table 4. Gene markers used for annotating each of the 26 major cell clusters.

Supplementary Table 5. Gene markers used for annotating each of cell types identified by sub-clustering on individual major cell clusters.

Supplementary Table 6. Significant genes which are (either positively or negatively) correlated with PC1-3 identified within NMPs & spinal cord progenitors and Mesoderm (Tbx6+) cells at the early somitogenesis.

Supplementary Table 7. Differential expressed genes identified from NMPs (T+, Meis1-) derived from earlier (0-12 somites) vs. later (14-34 somites) embryos.

Supplementary Table 8. Significant genes which are (either positively or negatively) correlated with PC1-3 identified within Notochord cells at the early somitogenesis.

Supplementary Table 9. Significant genes which are (either positively or negatively) correlated with PC1-3 identified within Gut cells at the early somitogenesis.

Supplementary Table 10. 198 genes which are significantly correlated to both PC1 of Notochord and PC1 of Gut at the early somitogenesis.

Supplementary Table 11. 82 genes which are significantly differentially expressed between early vs. late NMPs and correlated to PC2 of Gut at the early somitogenesis.

Supplementary Table 12. Gene markers used for annotating the sub cell types of different cell populations (e.g. neurogenesis).

Supplementary Table 13. Differential expressed genes identified from hepatic mesenchyme progenitors vs. foregut mesenchyme progenitors.

Supplementary Table 14. Top highly expressed TFs identified in each of the 15 sub-clusters of Retinal ganglion cells.

Supplementary Table 15. Top highly expressed TFs identified in each of the 11 spinal interneurons.

Supplementary Table 16. Significant genes which are (either positively or negatively) correlated with PC1-4 identified within the 11 spinal interneurons.

Supplementary Table 17. Datasets used for systematic reconstruction of the cellular trajectories of mouse embryogenesis.

Supplementary Table 18. Original cell types are regrouped into different developmental subsystems.

Supplementary Table 19. Some updates on cell-type annotations.

Supplementary Table 20. 283 distinct cell states identified across mouse embryogenesis.

Supplementary Table 21. Mutual nearest neighbor pairs identified within individual subsystems.

Supplementary Table 22. Edges after manually revising.

Supplementary Table 23. Systematic nomination of key transcription factors for cell type specification in mouse.

Supplementary Table 24. Systematic nomination of key genes for cell type specification in mouse.

Supplementary Table 25. Differentially expressed genes in the top 20 cell types at E18.75 vs. P0, ranked by degree of transcriptional disjunction between P0 and stages before P0, as shown in Figure 6b.

Supplementary Table 26. Significant genes which are differentially expressed across C-section timepoints in the validation dataset, in selected cell types from hepatocytes, adipocytes, and lung & airway.

Supplementary Table 27. Significant genes which are differentially expressed between three naturally birthed samples vs. C-section 20 & 40 mins in hepatocytes.

References

1. Centanin, L. & Wittbrodt, J. Retinal neurogenesis. *Development* **141**, 241–244 (2014).
2. Clark, B. S. *et al.* Single-Cell RNA-Seq Analysis of Retinal Development Identifies NFI Factors as Regulating Mitotic Exit and Late-Born Cell Specification. *Neuron* **102**, 1111–1126.e5 (2019).
3. Cepko, C. Intrinsically different retinal progenitor cells produce specific types of progeny. *Nat. Rev. Neurosci.* **15**, 615–627 (2014).
4. Shekhar, K., Whitney, I. E., Butrus, S., Peng, Y.-R. & Sanes, J. R. Diversification of multipotential postmitotic mouse retinal ganglion cell precursors into discrete types. *Elife* **11**, (2022).
