# Supplementary material for: A single-cell time-lapse of mouse prenatal development from gastrula to birth
Source: Nature. 2024 Feb 14;626(8001):1084–93. doi: 10.1038/s41586-024-07069-w (PMC10901739; doi:10.1038/s41586-024-07069-w)
Supplement: Supplementary file 2 — Reporting Summary [file 41586_2024_7069_MOESM2_ESM.pdf]

nature research

Corresponding author(s): Jay Shendure

Last updated by author(s): Nov 20, 2023

Reporting Summary

Nature Research wishes to improve the reproducibility of the work that we publish. This form provides structure for consistency and transparency in reporting. For further information on Nature Research policies, see our Editorial Policies and the Editorial Policy Checklist.

Statistics

For all statistical analyses, confirm that the following items are present in the figure legend, table legend, main text, or Methods section.

n/a	Confirmed	
☐	☒	The exact sample size (*n*) for each experimental group/condition, given as a discrete number and unit of measurement
☐	☒	A statement on whether measurements were taken from distinct samples or whether the same sample was measured repeatedly
☐	☒	The statistical test(s) used AND whether they are one- or two-sided<br>*Only common tests should be described solely by name; describe more complex techniques in the Methods section.*
☐	☒	A description of all covariates tested
☐	☒	A description of any assumptions or corrections, such as tests of normality and adjustment for multiple comparisons
☐	☒	A full description of the statistical parameters including central tendency (e.g. means) or other basic estimates (e.g. regression coefficient) AND variation (e.g. standard deviation) or associated estimates of uncertainty (e.g. confidence intervals)
☐	☒	For null hypothesis testing, the test statistic (e.g. *F*, *t*, *r*) with confidence intervals, effect sizes, degrees of freedom and *P* value noted<br>*Give P values as exact values whenever suitable.*
☒	☐	For Bayesian analysis, information on the choice of priors and Markov chain Monte Carlo settings
☒	☐	For hierarchical and complex designs, identification of the appropriate level for tests and full reporting of outcomes
☐	☒	Estimates of effect sizes (e.g. Cohen's *d*, Pearson's *r*), indicating how they were calculated

Our web collection on statistics for biologists contains articles on many of the points above.

Software and code

Policy information about availability of computer code

Data collection	No software was used except Illumina RTA basecalling at this stage.
Data analysis	The Python (version 3.10.10) and R (version 3.6.3 and 4.2.3) codes used to analyze the RNA-seq data are available at https://github.com/ChengxiangQiu/JAX_code. The following common, freely available data analysis software were used to analyze data: bcl2fastq version 2.20 (https://support.illumina.com), deML version 1.1.3 (https://github.com/grenaud/deML), HTseq version 0.6.1 (https://github.com/htseq/htseq), trim_galore version 0.6.5 (https://github.com/FelixKrueger/TrimGalore), STAR version 2.6.1d (https://github.com/alexdobin/STAR), scrublet version 0.1 (https://github.com/swolock/scrublet), Scanpy version 1.6.0 (https://github.com/theislab/scanpy), Monocle version 3, and 3-alpha (https://cole-trapnell-lab.github.io/monocle3), Seurat version 3 (https://github.com/satijalab/seurat), Tangram version 1.0.3 (https://github.com/broadinstitute/Tangram), Cytoscape version 3.9.1 (https://cytoscape.org/), geosketch version 1.2 (https://github.com/brianhie/geosketch).

For manuscripts utilizing custom algorithms or software that are central to the research but not yet described in published literature, software must be made available to editors and reviewers. We strongly encourage code deposition in a community repository (e.g. GitHub). See the Nature Research guidelines for submitting code & software for further information.

Data

Policy information about availability of data

All manuscripts must include a data availability statement. This statement should provide the following information, where applicable:

- Accession codes, unique identifiers, or web links for publicly available datasets
- A list of figures that have associated raw data
- A description of any restrictions on data availability

The data generated in this study can be downloaded in raw and processed forms from the NCBI Gene Expression Omnibus under accession number GSE186069 and

GSE228590. The data are also available at https://omg.gs.washington.edu/, together with a browser that enables its visual exploration. The published datasets analyzed for this study were retrieved from either the GEO repository (GSE44183, GSE100597, GSE109071), https://github.com/MarioniLab/EmbryoTimecourse2018, or https://db.cngb.org/stomics/mosta/ and re-processed. Published ISH images were obtained from the MGI website (https://www.informatics.jax.org/). Mouse reference genome (mm10) and gene annotations (GENCODE VM12) were used for read alignment and gene count matrix generation.

Field-specific reporting

Please select the one below that is the best fit for your research. If you are not sure, read the appropriate sections before making your selection.

☒ Life sciences ☐ Behavioural & social sciences ☐ Ecological, evolutionary & environmental sciences

For a reference copy of the document with all sections, see nature.com/documents/nr-reporting-summary-flat.pdf

Life sciences study design

All studies must disclose on these points even when the disclosure is negative.

Sample size

No statistical methods were used to predetermine sample size.
Our previous study (Qiu et al., 2022), which profiled 154,313 cells from 12 mouse embryos at early somitogenesis stage, successfully identified the same 30 cell types as those identified in E8.5 data by Pijuan-Sala et al. (2019). The extensive data, along with the separate processing of individual somite-resolved embryos, enabled the detection of significant substructures, such as A-P floor plates and various hindbrain segmentations. In this study, we applied the same technology to profile single nuclei from mouse embryos, identifying over 200 distinct cell types and focusing on several specific tissues and organs. This comprehensive analysis suggests that our sample size is adequate for investigating cell states and developmental trajectories during mouse organogenesis. In addition, we experimentally quantified the total DNA of staged embryos and estimated that the embryo grows 3,000-fold from E8.5 to P0. Therefore, despite the large number of nuclei profiled, our cellular coverage remains limited, ranging from 0.5-fold for early stages (summing 6 embryos, somite counts 7-12) to 0.002-fold immediately before birth (summing 6 embryos, E17.5-E18.75).

Data exclusions

When we took a first round of cell-embedding, we noticed that one mouse embryo at E14.5 had a grossly reduced proportion of neuronal cells. This particular sample had been divided during pulverization, and we suspect that large portions of the frozen embryo did not make it into the experiment. We removed cells from this E14.5 embryo.

Replication

Firstly, we performed 15 sci-RNA-seq3 experiments, and the data from each experiment overlapped well, demonstrating high replicability. We have employed various methods to confirm the data quality. Secondly, to validate our findings regarding posterior embryos, we generated an independent validation dataset comprising somites 8-21, and the findings were validated. Thirdly, to validate our observations of abrupt transcriptional changes before and after birth, we generated a new "birth-series" dataset, and the findings were validated. Finally, for the spatial mapping analysis, we utilized publicly available ISH images to verify our cell-type annotations within the lateral plate mesoderm.

Randomization

From a total of 523 embryos staged at the Jackson Laboratory, we selected 75 for whole embryo scRNA-seq, targeting one embryo for every somite count from 0 to 34 (2-hr increments), and one embryo for every 6-hr bin from E10 to P0. Embryos used in experiments were randomly selected from each timepoint before sample preparation.

Blinding

In this study, investigators were blinded to group allocation during sample collection and data generation/analysis: embryo collection and sci-RNA-seq3 data generation/analysis were performed by different researchers in different locations.

Reporting for specific materials, systems and methods

We require information from authors about some types of materials, experimental systems and methods used in many studies. Here, indicate whether each material, system or method listed is relevant to your study. If you are not sure if a list item applies to your research, read the appropriate section before selecting a response.

Materials & experimental systems

n/a	Involved in the study	
☒	☐	Antibodies
☒	☐	Eukaryotic cell lines
☒	☐	Palaeontology and archaeology
☐	☒	Animals and other organisms
☒	☐	Human research participants
☒	☐	Clinical data
☒	☐	Dual use research of concern

Methods

n/a	Involved in the study	
☒	☐	ChIP-seq
☒	☐	Flow cytometry
☒	☐	MRI-based neuroimaging

Animals and other organisms

Policy information about studies involving animals; ARRIVE guidelines recommended for reporting animal research

Laboratory animals	83 precisely staged C57BL/6NJ (strain# 005304) mice were obtained at The Jackson Laboratory. Mice of both sexes were included in the study, with a roughly equal number of males and females.
Wild animals	Study did not involve wild animals
Field-collected samples	Study did not involve field-collected samples
Ethics oversight	All animal use at The Jackson Laboratory was done in accordance with the Animal Welfare Act and the AVMA Guidelines on Euthanasia, in compliance with the ILAR Guide for Care and Use of Laboratory Animals, and with prior approval from The Jackson Laboratory animal care and use committee (ACUC) under protocol AUS20028.

Note that full information on the approval of the study protocol must also be provided in the manuscript.
